# Identification of postoperative weight loss trajectories and development of a machine learning-based tool for predicting malnutrition in gastric cancer patients

**DOI:** 10.3389/fnut.2025.1678879

**Published:** 2025-09-17

**Authors:** Mingfang Yan, Zhenmeng Lin, Rong Chen, Ying Liu, Jinliang Jian, Changhua Zhuo

**Affiliations:** ^1^Department of Anesthesiology Surgery, Clinical Oncology School of Fujian Medical University, Fujian Cancer Hospital, Fuzhou, Fujian, China; ^2^Department of Gastrointestinal Surgery, Clinical Oncology School of Fujian Medical University, Fujian Cancer Hospital, Fuzhou, Fujian, China; ^3^College of Animal Science, Fujian Agriculture and Forestry University, Fuzhou, Fujian, China

**Keywords:** gastric cancer, weight loss trajectories, malnutrition, machine learning, nomogram

## Abstract

**Background:**

Significant postoperative weight loss and malnutrition represent common and serious complications following radical gastrectomy for gastric cancer. Early identification of distinct weight loss trajectories and prediction of malnutrition risk may facilitate targeted interventions.

**Methods:**

This prospective, observational longitudinal study enrolled 312 gastric adenocarcinoma patients undergoing radical gastrectomy. Participants were assessed preoperatively (T0) and at 3, 6, 9, and 12 months postoperatively (T1–T4). Percentage weight loss was calculated at each postoperative time point. Latent growth mixture modeling (GMM) identified distinct weight loss trajectories. Eight machine learning algorithms (XGBoost, SVM, RF, NB, KNN, MLP, GBM, PLS) were trained using predictors selected by LASSO regression and the Boruta algorithm to predict GLIM-defined malnutrition at 6 months postoperatively (T2, the peak malnutrition timepoint). Additionally, a multivariable logistic regression-derived nomogram was developed and validated, with assessments of discrimination, calibration, and clinical utility.

**Results:**

GMM identified three distinct 12-month postoperative weight loss trajectories: severe (11.9%), moderate (36.2%), and minimal (51.9%). The prevalence of GLIM-defined malnutrition peaked at 51.6% at 6 months (T2). Among the eight machine learning models, XGBoost achieved the best performance in predicting 6-month malnutrition. The final nomogram, which incorporated age ≥65 years, preoperative underweight status, preoperative reduced muscle mass, and total gastrectomy, showed excellent discrimination, calibration, and clinical utility. DeLong’s test indicated no significant difference in AUC between the XGBoost model and the nomogram (*p* = 0.121).

**Conclusion:**

This study delineates distinct postoperative weight loss trajectories in gastric cancer patients. We developed and validated both an advanced ML model (XGBoost) and a clinically interpretable nomogram for accurately predicting 6-month postoperative malnutrition risk.

## 1 Introduction

Gastric cancer remains a major global health burden, accounting for the fifth most common malignancy and the fourth leading cause of cancer-related mortality worldwide (1). Although radical gastrectomy offers curative potential, it causes profound physiological alterations, including reduced gastric capacity, dysregulation of digestive hormones, impaired digestive function, and malabsorption (2–4). These mechanisms collectively lead to substantially high postoperative malnutrition rates, affecting 40–60% of patients within the first year (2, 5, 6), establishing malnutrition as a prevalent yet underrecognized complication among gastric cancer survivors.

Importantly, postoperative malnutrition predicts devastating clinical outcomes beyond symptomatic concerns. Robust evidence links it to increased chemotherapy toxicity, heightened infection risk, elevated readmission rates, diminished quality of life, and reduced overall survival (7–13). Consequently, early identification of high-risk patients is essential for implementing timely nutritional interventions proven to mitigate these sequelae (14–16).

Postoperative malnutrition in gastric cancer patients is a critical issue, yet current predictive tools have significant limitations, including reliance on cross-sectional data that overlook dynamic nutritional changes, limited generalizability from small single-center studies, inter-observer variability in subjective assessments, and neglect of key factors such as surgical extent and preoperative body composition. Complex models often lack clinical practicality due to poor interpretability (2, 6, 17, 18). While machine learning has been used for malnutrition risk prediction and trajectory modeling in other cancer or surgical populations (e.g., post-bariatric surgery or broader oncology cohorts), these approaches rarely integrate to capture the heterogeneous recovery patterns specific to gastric cancer survivorship (19–21). To address these gaps, our study combines latent growth mixture modeling (GMM) with machine learning to characterize 12-month postoperative weight loss trajectories and develop a robust, clinically applicable tool for predicting malnutrition using key preoperative and perioperative predictors. This approach enables precision nutritional management by identifying high-risk patients early.

## 2 Methods

### 2.1 Study design and participants

This prospective longitudinal observational study consecutively enrolled patients undergoing gastric cancer surgery at Fujian Cancer Hospital between January 2023 and May 2024. Participants were assessed at five predefined time points: preoperatively (T0), and 3, 6, 9, and 12 months postoperatively (T1-T4). Inclusion criteria included: (1) histologically confirmed gastric adenocarcinoma; (2) radical gastrectomy; and (3) provision of written informed consent. Exclusion criteria were: (1) concurrent malignancies; (2) severe cardiac, hepatic, pulmonary, or renal impairment; or (3) cognitive impairment or psychiatric disorders compromising reliable communication. Patients with tumor recurrence/metastasis or mortality during the 12-month follow-up were excluded from trajectory analysis.

### 2.2 Measurements and definition

Percentage weight loss (%) was calculated as [(preoperative weight – postoperative weight)/preoperative weight] × 100, and assessed at all postoperative time points (T1–T4).

Axial computed tomography (CT) scans at the third lumbar vertebra (L3) level were analyzed using SliceOmatic software (version 5.0; TomoVision, Montreal, QC, Canada). Skeletal muscle area (SMA, cm^2^) was quantified by identifying muscle tissue (Supplementary Figure 1). The skeletal muscle index (SMI, cm^2^/m^2^) was subsequently derived by normalizing SMA to height squared (SMA/height^2^). Reduced muscle mass was defined according to established cut-offs: <34.9 cm^2^/m^2^ for females and <40.8 cm^2^/m^2^ for males (22–24).

Nutritional risk was screened using the Nutritional Risk Screening 2002 (NRS-2002), with scores ≥3 indicating a risk of malnutrition. Malnutrition was diagnosed according to the Global Leadership Initiative on Malnutrition (GLIM) criteria, which require: (1) ≥1 phenotypic criterion, including non-volitional weight loss (>5% within 6 months or >10% beyond 6 months), low BMI [<18.5 kg/m^2^ for age <70 years or <20 kg/m^2^ for age ≥70 years], or CT-defined reduced muscle mass; and (2) ≥1 etiologic criterion (25, 26). Given the chronic inflammatory nature of malignancy, all patients were considered automatically to satisfy the etiologic criterion (inflammation/disease burden) (25, 27, 28). Assessments were conducted at preoperative (T0), 6-month (T2), and 12-month (T4) time points, synchronized with institutional CT imaging protocols.

Anemia was defined as hemoglobin levels <120 g/L in adult males and <110 g/L in non-pregnant adult females (29). Hypoalbuminemia was defined as serum albumin levels <35 g/L, a threshold associated with adverse clinical outcomes in patients with gastrointestinal cancer (30). Body mass index (BMI) was categorized as follows: underweight (<18.5 kg/m^2^), normal weight (18.5 to <24 kg/m^2^), and overweight (≥24 kg/m^2^) (31, 32). Postoperative complications were classified by severity using the Clavien-Dindo classification system, with Grade III or higher defined as major complications (33, 34).

### 2.3 Statistical analysis

All analyses were performed using R (version 4.4.2), Mplus (version 7.4), and SPSS (version 24.0). Categorical variables were compared using chi-square tests, while normally distributed continuous variables were analyzed using one-way analysis of variance (ANOVA). Longitudinal changes in BMI were assessed using linear mixed-effects models (LMMs). Percentage weight loss trajectories were identified via GMM. Model fit was evaluated using the following indices: Akaike Information Criterion (AIC), Bayesian Information Criterion (BIC), adjusted BIC (aBIC), entropy, Lo–Mendell–Rubin Likelihood Ratio Test (LMR-LRT), and Bootstrap Likelihood Ratio Test (BLRT). Predictors of trajectory class membership were determined using multivariable logistic regression.

For predicting 6-month postoperative malnutrition, feature selection was conducted using Least Absolute Shrinkage and Selection Operator (LASSO) regression and the Boruta algorithm, followed by the implementation of eight machine learning algorithms: eXtreme Gradient Boosting (XGBoost), Support Vector Machine (SVM), Random Forest (RF), Naïve Bayes (NB), K-Nearest Neighbors (KNN), Multilayer Perceptron (MLP), Gradient Boosting Machine (GBM), and Partial Least Squares (PLS). All models were trained and evaluated using 10-fold cross-validation, with all reported performance metrics representing the average across the validation folds to ensure generalizability. Feature importance was interpreted using SHapley Additive exPlanations (SHAP). Independent risk factors for malnutrition were identified via multivariable logistic regression to construct a nomogram. The nomogram was validated using the area under the receiver operating characteristic curve (AUC-ROC), calibration curves with the Hosmer-Lemeshow test, and decision curve analysis (DCA). Statistical significance was defined as a two-tailed *p* < 0.05.

## 3 Results

### 3.1 Participant characteristics

**Figure 1:**
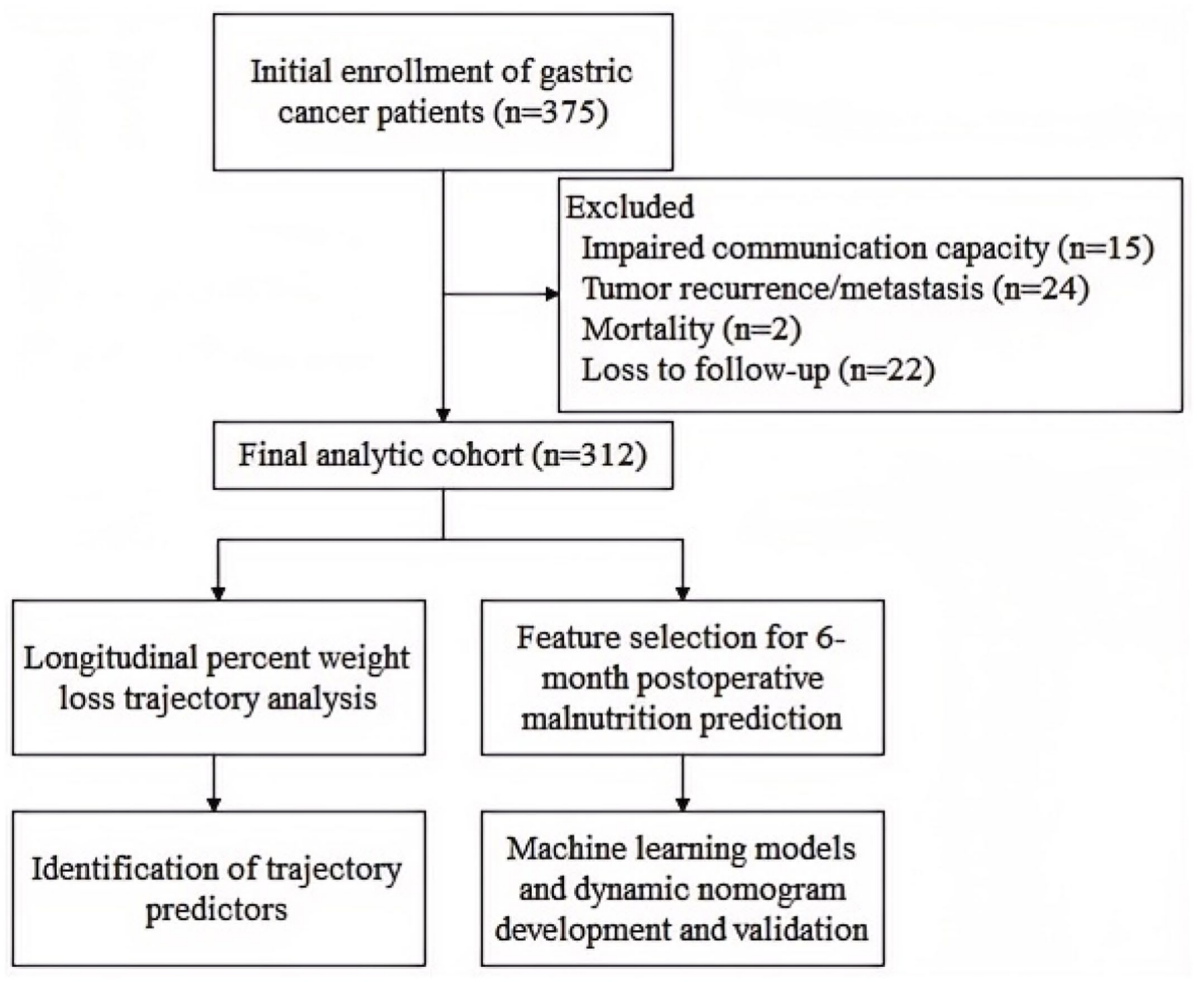
Flowchart of the longitudinal trajectory-to-prediction framework.

A total of 375 gastric cancer patients who underwent radical gastrectomy were initially enrolled. Of these, 63 patients were excluded due to: tumor recurrence/metastasis, mortality, loss to follow-up, or impaired communication capacity. Ultimately, 312 patients who completed at least three follow-up surveys were included in the final analysis (Figure 1).

Statistically significant differences in percentage weight loss were observed at 3, 6, 9, and 12 months postoperatively (*p* < 0.05), as detailed in Supplementary Figure 2.

### 3.2 Identification of distinct postoperative weight loss trajectories

**Figure 2:**
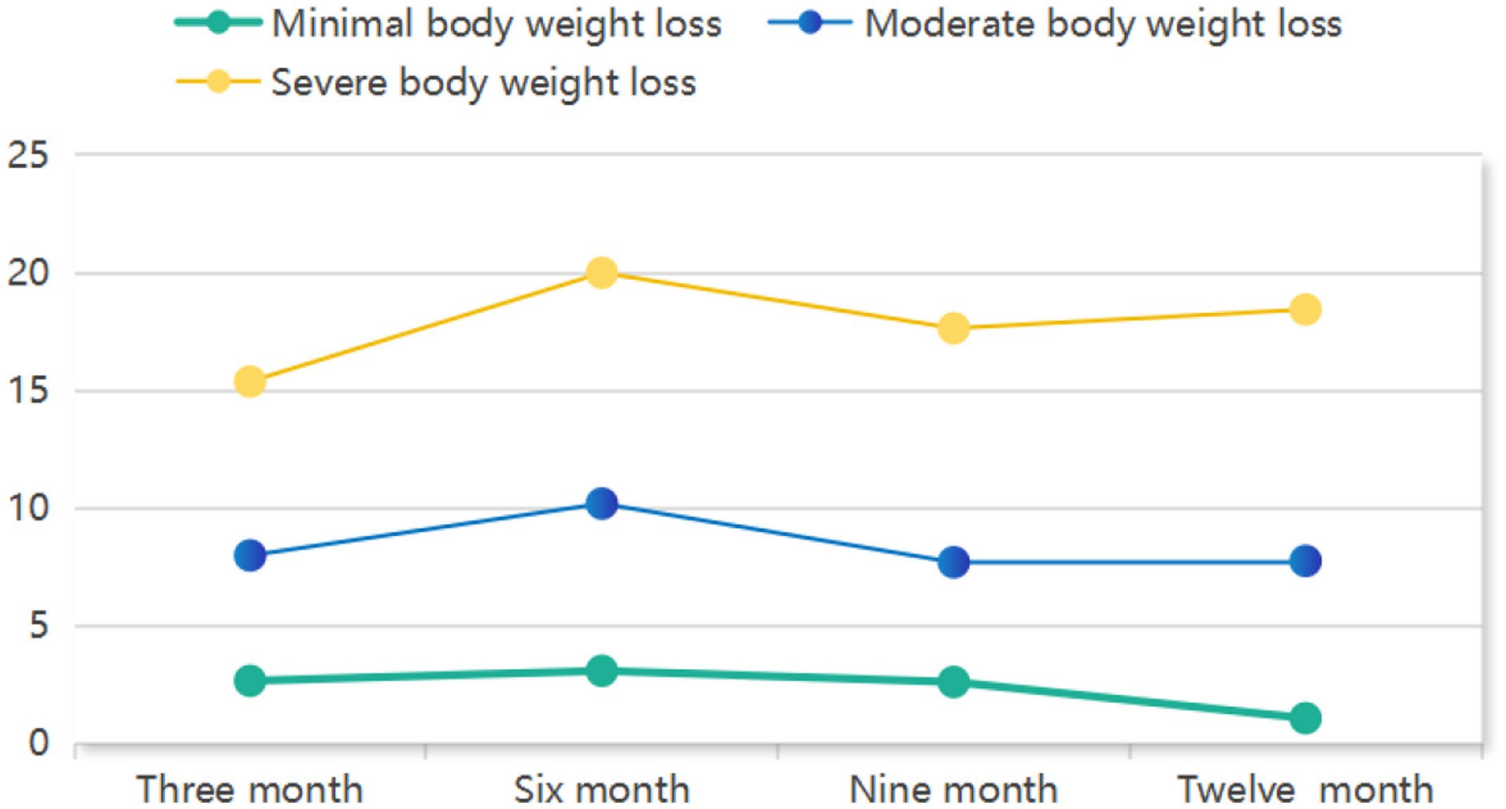
Three distinct postoperative weight loss trajectories.

GMM identified three distinct trajectories of postoperative weight loss as the optimal solution (Table 1). The 3-class model demonstrated superior fit indices, including the lowest AIC (5181.058), BIC (5267.147) and aBIC (5194.199) values, statistically significant LMR-LRT (*p* < 0.001) and BLRT (*p* < 0.001), and high entropy (0.989). Clinically, these trajectories were defined as: Severe weight loss (11.9%, *n* = 37), Moderate weight loss (36.2%, *n* = 113), and Minimal weight loss (51.9%, *n* = 162). As visually depicted in Figure 2, these trajectory groups exhibited significantly divergent percentage weight loss patterns throughout the 12-month postoperative period (*p* < 0.001 by LMMs).

**Table 1:** Growth mixture model fit statistics for weight loss trajectories.

Classes	AIC	BIC	aBIC	Category	*p-*value		Category percentage (%)
					LMRT	BLRT	
1	7702.469	7732.413	7707.040	–	–	–	–
2	6645.160	6693.819	6652.587	0.988	0.0027	0.0031	0.859/0.141
3	5181.058	5267.147	5194.199	0.989	0.000	0.000	0.519/0.361/0.120
4	5615.692	5683.066	5625.976	0.975	0.3223	0.3308	0.508/0.352/0.074/0.067
5	5845.868	5950.672	5861.866	0.973	0.2222	0.2325	0.470/0.171/0.222/0.066/0.071

### 3.3 Comparative analysis of predictive determinants across weight loss trajectory groups

Univariate analyses revealed significant differences across the three weight loss trajectory groups in several clinicopathological characteristics: age, sex, BMI, type of operation, pathological stage, postoperative major complications, and adjuvant chemotherapy (Table 2).

**Table 2:** Univariate analysis of clinicopathological characteristics by weight loss trajectory group.

Characteristics	Severe weight loss (*n* = 37)	Moderate weight loss (*n* = 113)	Minimal weight loss (*n* = 162)	*P*
Age, year				0.008
<65	13 (35.1)	65 (57.5)	102 (63.0)	
≥65	24 (64.9)	48 (42.5)	60 (37.0)	
Sex				0.019
Male	19 (51.4)	64 (56.6)	114 (70.4)	
Female	18 (48.6)	49 (43.4)	48 (29.6)	
Marital status				0.390
Spouse	26 (70.3)	91 (80.5)	129 (79.6)	
No spouse	11 (29.7)	22 (19.5)	33 (20.4)	
Family history				0.441
Yes	8 (21.6)	19 (16.8)	22 (13.6)	
No	29 (78.4)	94 (83.2)	140 (86.4)	
Preoperative BMI, kg/m^2^				0.005
Underweight	3 (8.1)	20 (17.7)	41 (25.3)	
Normal	16 (43.2)	66 (58.4)	85 (52.5)	
Overweight	18 (48.6)	27 (23.9)	36 (22.2)	
Smoking history				0.446
Yes	8 (21.6)	22 (19.5)	42 (25.9)	
No	29 (78.4)	91 (80.5)	120 (74.1)	
Alcohol consumption				0.226
Yes	15 (40.5)	29 (25.7)	48 (29.6)	
No	22 (59.5)	84 (74.3)	114 (70.4)	
Previous abdominal surgery				0.535
Yes	5 (13.5)	12 (10.6)	13 (8.0)	
No	32 (86.5)	101 (89.4)	149 (92.0)	
Hypertension				0.268
Yes	15 (40.5)	31 (27.4)	45 (27.8)	
No	22 (59.5)	82 (72.6)	117 (72.2)	
Diabetes mellitus				0.292
Yes	11 (29.7)	20 (17.7)	35 (21.6)	
No	26 (70.3)	93 (82.3)	127 (78.4)	
Dyslipidemia				0.102
Yes	14 (37.8)	26 (23.0)	54 (33.3)	
No	23 (62.2)	87 (77.0)	108 (66.7)	
Preoperative anemia				0.336
Yes	15 (40.5)	33 (29.2)	46 (28.4)	
No	22 (59.5)	80 (70.8)	116 (71.6)	
Preoperative hypoalbuminemia				0.596
Yes	15 (40.5)	36 (31.9)	58 (35.8)	
No	22 (59.5)	77 (68.1)	104 (64.2)	
Preoperative reduced muscle mass				0.255
Yes	9 (24.3)	22 (19.5)	23 (14.2)	
No	28 (75.7)	91 (80.5)	139 (85.8)	
CEA, ng/ml				0.403
<5	29 (78.4)	89 (78.8)	137 (84.6)	
≥5	8 (21.6)	24 (21.2)	25 (15.4)	
CA19-9, U/ml				0.257
<30	29 (78.4)	93 (82.3)	142 (87.7)	
≥30	8 (21.6)	20 (17.7)	20 (12.3)	
ASA grade				0.098
I-II	35 (94.6)	93 (82.3)	144 (88.9)	
III-IV	2 (5.4)	20 (17.7)	18 (11.1)	
Tumor size, cm	2.72 ± 1.20	2.44 ± 1.04	2.53 ± 1.00	0.350
Operation method				0.102
Open	17 (45.9)	33 (29.2)	46 (28.4)	
Laparoscopy	20 (54.1)	80 (70.8)	116 (71.6)	
Type of operation				0.007
Distal gastrectomy	15 (40.5)	78 (69.0)	105 (64.8)	
Total gastrectomy	22 (59.5)	35 (31.0)	57 (35.2)	
Operation time (h)				0.342
<4	21 (56.8)	78 (69.0)	102 (63.0)	
≥4	16 (43.2)	35 (31.0)	60 (37.0)	
Estimated blood loss (ml)				0.387
<200	22 (59.5)	56 (49.6)	76 (46.9)	
≥200	15 (40.5)	57 (50.4)	86 (53.1)	
Histological type				0.497
Well/Moderately	6 (16.2)	29 (25.7)	39 (24.1)	
Poorly/Undifferentiated	31 (83.8)	84 (74.3)	123 (75.9)	
Perioperative blood transfusion				0.505
Yes	9 (24.3)	18 (15.9)	31 (19.1)	
No	28 (75.7)	95 (84.1)	131 (80.9)	
Number of removed lymph nodes, mean (SD)	33.6 ± 5.5	34.4 ± 11.2	35.7 ± 9.1	0.348
Pathological stage				0.021
I	9 (24.3)	24 (21.2)	48 (29.6)	
II	8 (21.6)	14 (12.4)	39 (24.1)	
III	20 (54.1)	75 (66.4)	75 (46.3)	
Postoperative major complications				0.021
Yes	7 (18.9)	21 (18.6)	13 (8.0)	
No	30 (81.1)	92 (81.4)	149 (92.0)	
Adjuvant chemotherapy				0.028
Yes	31 (83.8)	68 (60.2)	110 (67.9)	
No	6 (16.2)	45 (39.8)	52 (32.1)	

Multivariable logistic regression analyses comparing pairs of trajectory groups (Table 3) identified distinct predictors associated with group membership. Compared to the severe weight loss group, membership in the moderate weight loss group was significantly predicted by: younger age (OR = 0.412, 95%CI: 0.184–0.926; *p* = 0.032), lower BMI (OR = 0.474, 95%CI: 0.251–0.896; *p* = 0.022), undergoing distal gastrectomy (vs. total) (OR = 0.326, 95%CI: 0.145–0.731; *p* = 0.007), and no adjuvant chemotherapy (OR = 0.304, 95%CI: 0.113–0.817; *p* = 0.018). When compared to the minimal weight loss group, membership in the severe weight loss group was significantly associated with: older age (OR = 3.074, 95%CI: 1.394–6.779; *p* = 0.005), higher BMI (OR = 2.589, 95%CI: 1.389–4.824; *p* = 0.003), and undergoing total gastrectomy (vs. distal) (OR = 2.873, 95%CI: 1.306–6.320; *p* = 0.009). Finally, relative to the minimal weight loss group, membership in the moderate weight loss group was significantly associated with: female sex (OR = 0.513, 95%CI: 0.303–0.867; *p* = 0.013), advanced pathological stage (OR = 1.589, 95%CI: 1.169–2.158; *p* = 0.003), and presence of major complications (OR = 2.645, 95%CI: 1.233–5.682; *p* = 0.013).

**Table 3:** Multivariable logistic regression for pairwise trajectory group comparisons.

Variable	Class 1 vs. Class 2		Class 1 vs. Class 3		Class 2 vs. Class 3	
	OR (95%CI)	P	OR (95%CI)	P	OR (95%CI)	P
Age	0.412(0.184–0.926)	0.032	3.074 (1.394–6.779)	0.005	1.268(0.759–2.118)	0.365
Sex	0.958(0.431–2.129)	0.916	2.034(0.926–4.469)	0.077	1.949(1.153–3.296)	0.013
BMI	0.474(0.251–0.896)	0.022	2.589(1.389–4.824)	0.003	1.226(0.842–1.786)	0.287
Type of operation	0.326(0.145–0.731)	0.007	2.873(1.306–6.320)	0.009	0.936(0.546–1.604)	0.809
Pathological stage	1.208(0.746–1.954)	0.442	1.315(0.831–2.082)	0.242	1.589(1.169–2.158)	0.003
Postoperative major complications	0.965(0.335–2.785)	0.948	0.391(0.128–1.193)	0.099	0.378(0.176–0.811)	0.013
Adjuvant chemotherapy	3.294(1.224–8.863)	0.018	0.464(0.174–1.239)	0.125	1.528(0.903–2.585)	0.114

Class 1, Severe Weight Loss; Class 2, Moderate Weight Loss; Class 3, Minimal Weight Loss.

### 3.4 Longitudinal malnutrition prevalence and model timepoint selection

The prevalence of GLIM-defined malnutrition exhibited significant temporal variation: 18.6% (58/312) at T0, peaking at 51.6% (161/312) at T2, and declining to 40.7% (127/312) at T4. Consistent with this pattern, maximal postoperative percentage weight loss occurred at T2. Given this critical nutritional deterioration phase, T2 was selected for predictive model development.

### 3.5 Feature selection and machine learning model performance

**Figure 3:**
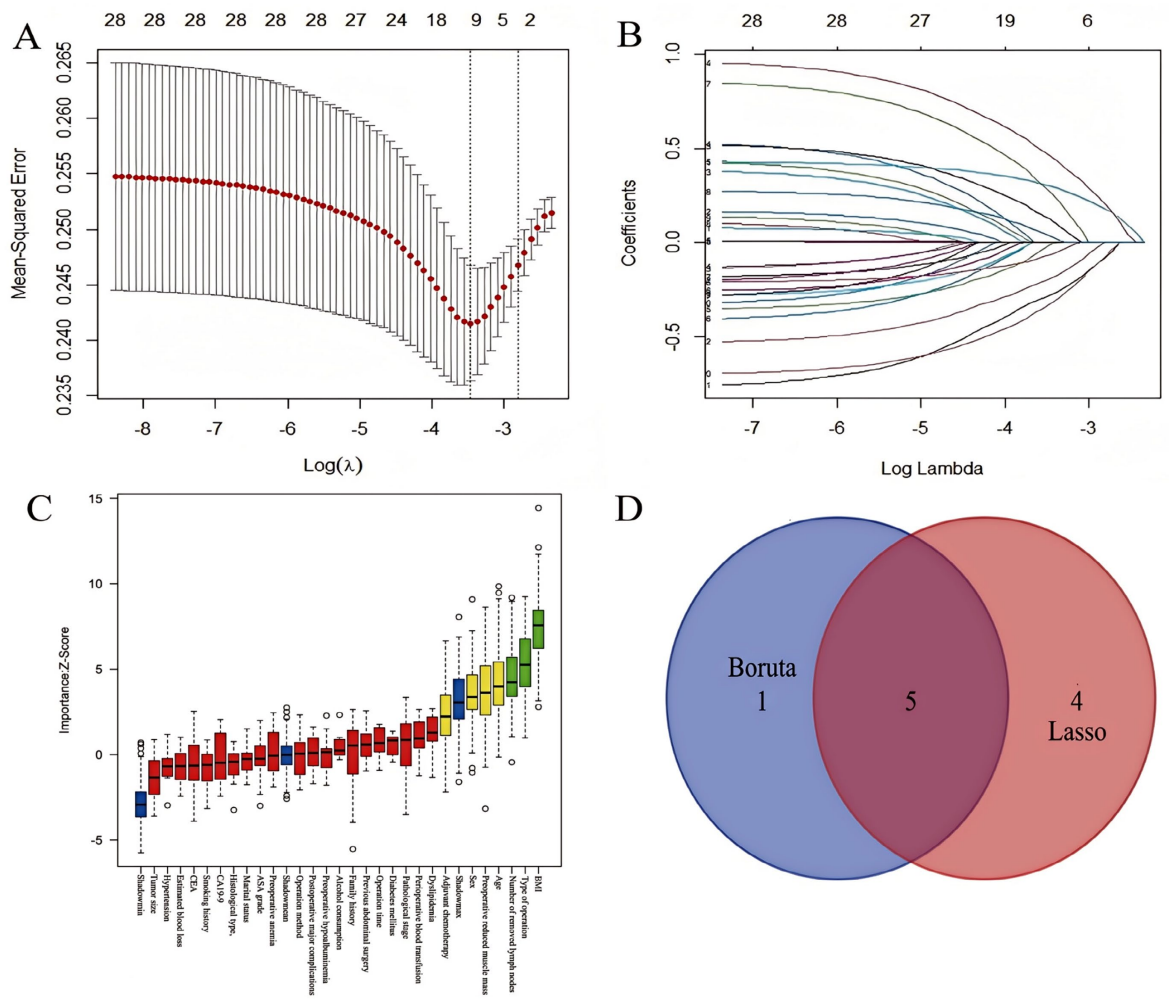
Feature selection process for predicting 6-month postoperative malnutrition. **(A)** LASSO coefficient profiles across log(*λ*) values; **(B)** Variables retained by LASSO at the optimal λ; **(C)** 6 key feature variables screened by the Boruta algorithm; **(D)** Intersection of features screened by LASSO regression and Boruta algorithm.

Feature selection was performed using the Boruta algorithm and LASSO regression to identify key predictors of GLIM-defined malnutrition at T2. The Boruta algorithm confirmed 6 features as significant predictors, while LASSO regression selected 9 variables as non-zero coefficients at the optimal lambda (*λ*). The intersection of features identified as significant by both methods yielded 5 consensus predictors: Sex, Age, Preoperative BMI, Preoperative reduced muscle mass, and Type of operation (Figure 3).

These five consensus predictors were used to train eight machine learning models for predicting GLIM-defined malnutrition at T2. Performance metrics---including sensitivity, specificity, Youden’s index, accuracy, negative predictive value (NPV), precision, recall, and F1-score---were evaluated using 10-fold cross-validation. Among all evaluated algorithms, XGBoost demonstrated superior overall predictive performance. It achieved the highest area under the receiver operating characteristic curve (AUC) of 0.855 (95% CI: 0.814–0.896), along with the highest specificity (0.921), precision (0.896), Youden’s index (0.560), accuracy (0.776), and F1-score (0.747) (Table 4 and Supplementary Figure 3).

**Table 4:** Comparative performance of machine learning models for predicting 6-month postoperative malnutrition.

Group	Specificity	Youden	Accuracy	Npv	Precision	Recall	F1	AUC
XGBoost	0.921	0.560	0.776	0.706	0.896	0.640	0.747	0.855(0.814–0.896)
SVM	0.808	0.510	0.753	0.718	0.796	0.702	0.746	0.821(0.776–0.866)
RF	0.861	0.339	0.663	0.607	0.786	0.478	0.594	0.735(0.681–0.790)
NB	0.808	0.386	0.689	0.642	0.762	0.578	0.657	0.720(0.663–0.776)
KNN	0.722	0.355	0.676	0.649	0.708	0.634	0.669	0.748(0.695–0.801)
MLP	0.795	0.385	0.689	0.645	0.754	0.590	0.662	0.756(0.703–0.809)
GBM	0.934	0.456	0.721	0.647	0.894	0.522	0.659	0.764(0.711–0.817)
PLS	0.954	0.401	0.692	0.618	0.911	0.447	0.600	0.763(0.711–0.815)

**Figure 4:**
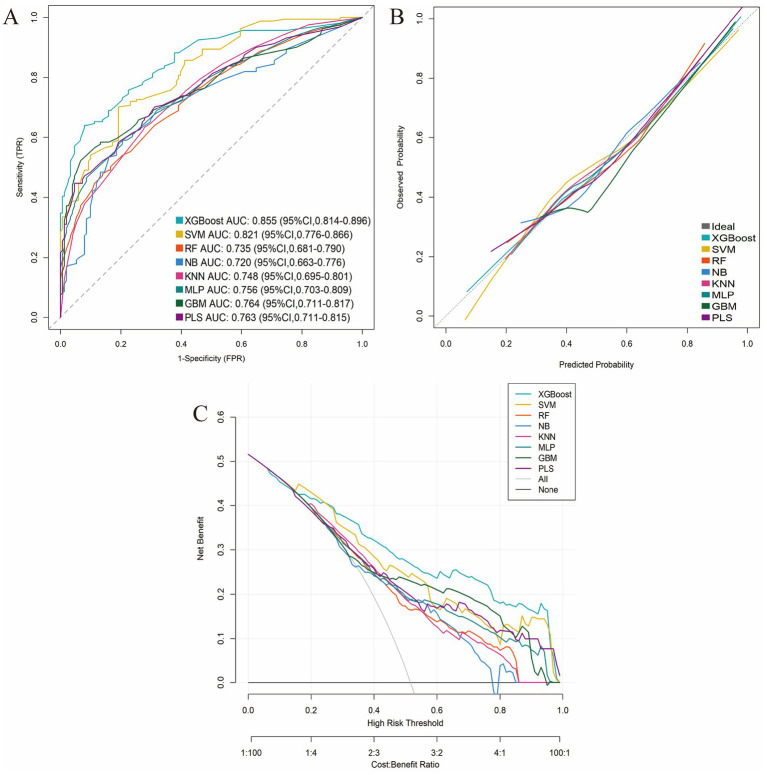
Comparative performance evaluation of the eight machine-learning models. **(A)** ROC curves; **(B)** Calibration plots; **(C)** DCA curves.

The superior performance of the XGBoost model was further substantiated through comprehensive internal validation: (1) The ROC curve confirmed its robust discriminative ability; (2) The calibration curve demonstrated excellent agreement between predicted probabilities and observed outcomes; and (3) DCA revealed significantly greater net clinical benefit across a wide range of clinically relevant threshold probabilities compared to the other machine learning models evaluated (Figure 4).

**Figure 5:**
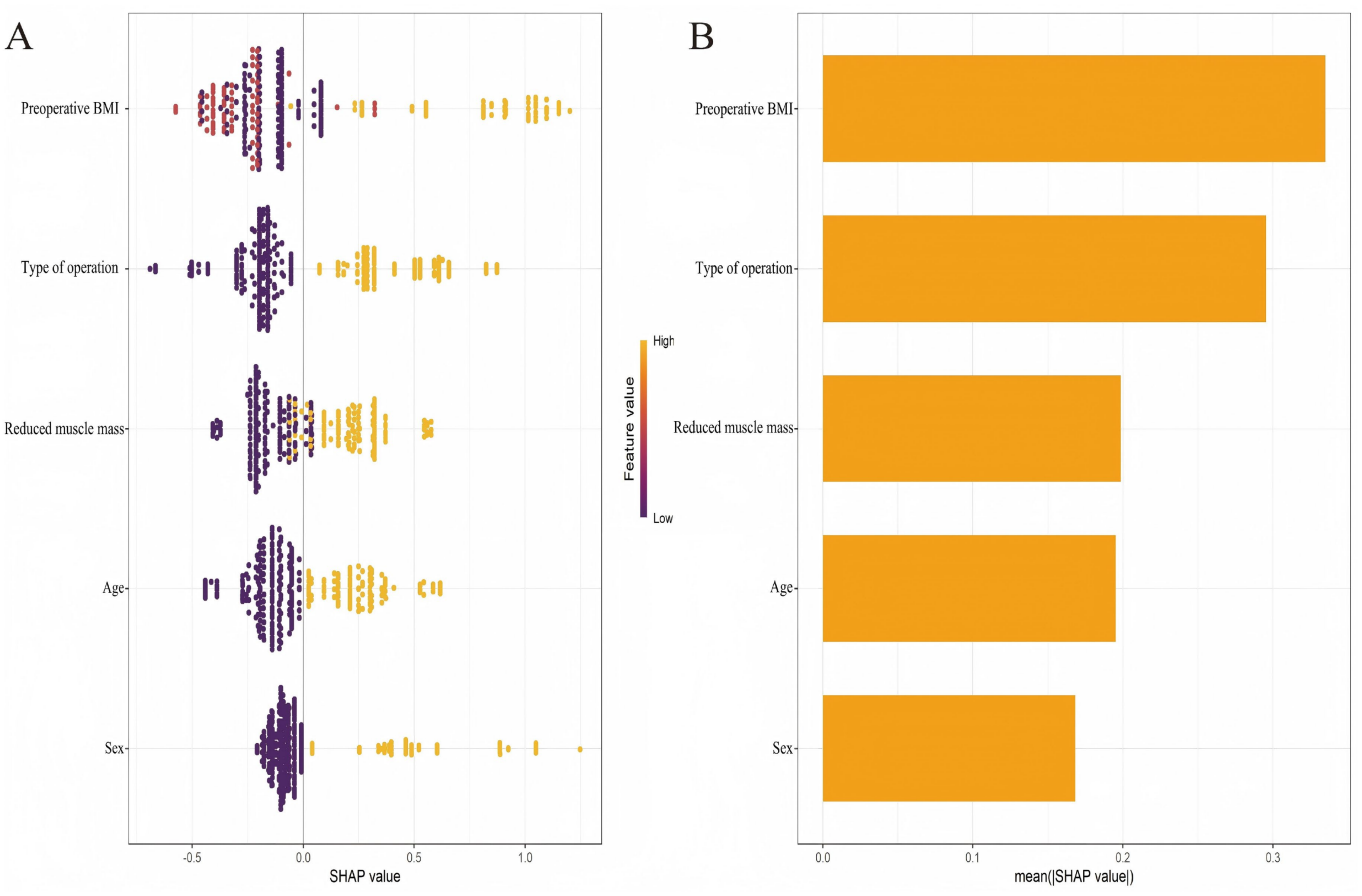
SHAP-based interpretability of the 6-month postoperative malnutrition prediction model. **(A)** Beeswarm plot of individual SHAP values. **(B)** Bar plot of mean absolute SHAP values.

Subsequently, SHAP analysis was performed to elucidate the contribution of each predictor within the XGBoost model. Based on the mean absolute SHAP values, the predictors were ranked in descending order of importance as follows: preoperative BMI, type of operation, preoperative reduced muscle mass, age, and sex (Figure 5).

### 3.6 Independent risk factors and nomogram construction for 6-month postoperative malnutrition

**Figure 6:**
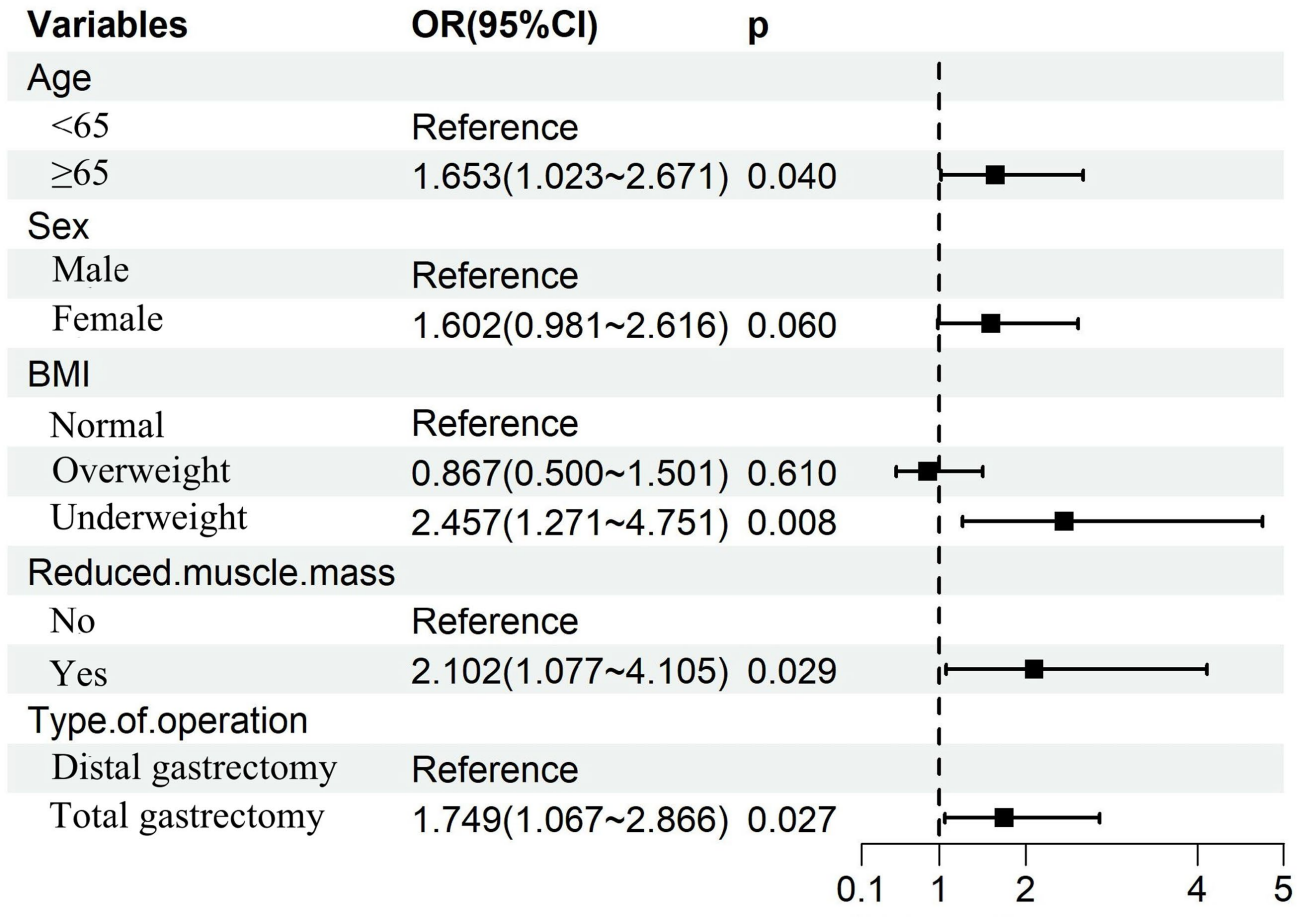
Forest plot of independent predictors for 6-month postoperative malnutrition.

**Figure 7:**
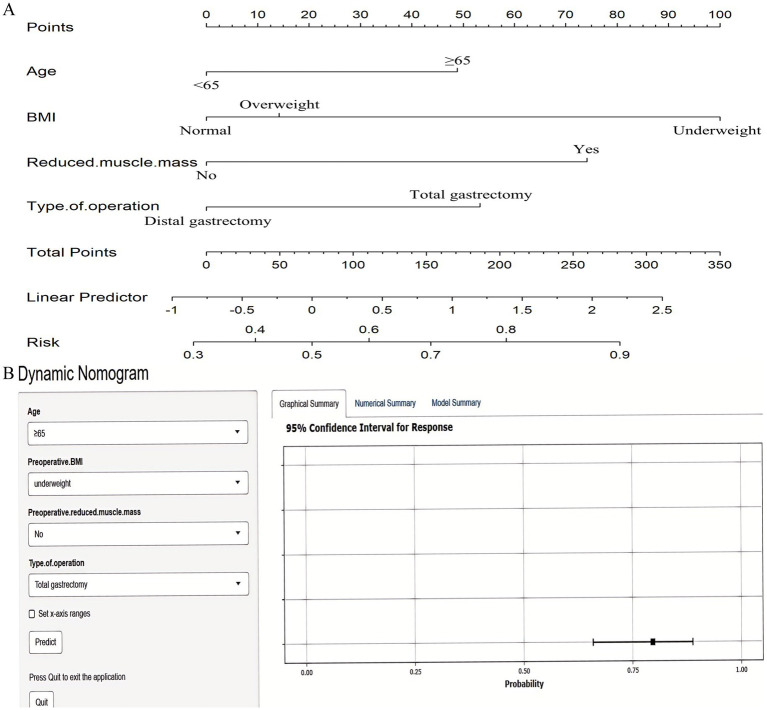
Nomograms for predicting 6-month postoperative malnutrition. **(A)** Static nomogram; **(B)** Dynamic nomogram.

Multivariable logistic regression analysis that incorporating the five consensus predictors derived from the intersection of LASSO and Boruta feature selection identified four independent risk factors for GLIM-defined malnutrition at 6 months postoperatively: Age ≥65 years; Preoperative underweight status; Preoperative reduced muscle mass; Total gastrectomy (Figure 6). Based on these four independent risk factors, a nomogram was developed to quantify individualized probabilities of GLIM-defined malnutrition at T2 (Figure 7A). To enhance clinical utility and facilitate point-of-care application, an interactive dynamic version of this nomogram was developed and is publicly accessible online^1^ (Figure 7B).

### 3.7 Validation of the nomogram for predicting 6-month postoperative malnutrition

**Figure 8:**
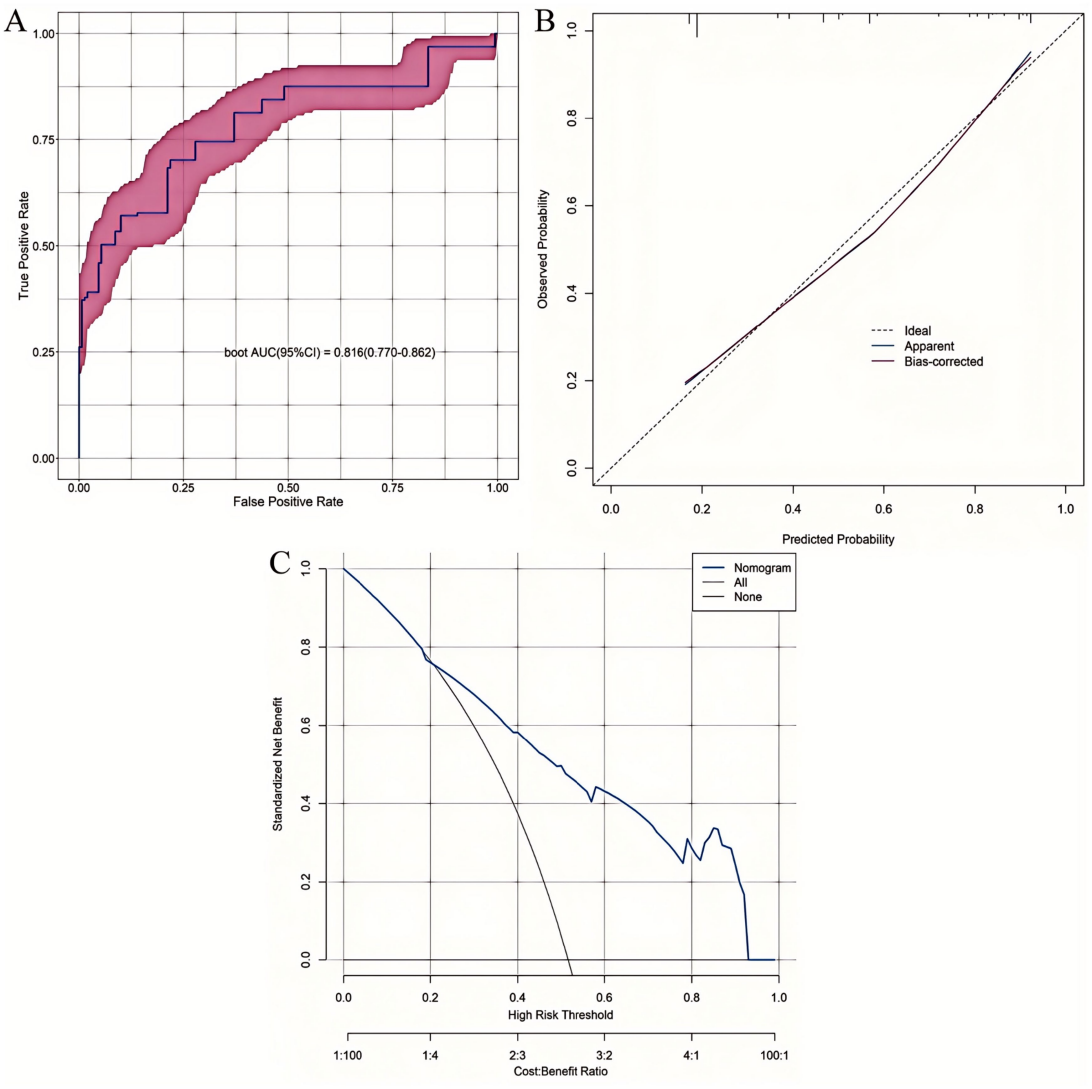
Comprehensive validation of the 6-month postoperative malnutrition nomogram. **(A)** ROC curve. **(B)** Calibration plot. **(C)** DCA curve.

The nomogram demonstrated strong discriminative ability for predicting 6-month postoperative malnutrition, with AUC of 0.816 (95% CI: 0.770–0.862) (Figure 8A). Calibration curves indicated excellent agreement between the nomogram-predicted probabilities of malnutrition and the observed frequencies (Figure 8B). DCA demonstrated superior clinical utility of the nomogram across a wide range of clinically relevant threshold probabilities (20 to 92%), showing a greater net benefit compared to strategies of intervening in all patients (“treat-all”) or no patients (“treat-none”) (Figure 8C).

### 3.8 Comparative discriminative performance: XGBoost vs. nomogram

To assess the comparative discriminative performance and potential clinical added value of the nomogram relative to the best-performing machine learning model, we compared the ROC curves of the XGBoost model and the nomogram using DeLong’s test for the equality of AUCs. DeLong’s test revealed no statistically significant difference in AUCs (*Z* = 1.549; *p* = 0.121), indicating that the nomogram achieved statistically equivalent discriminative power to the more complex XGBoost model.

## 4 Discussion

In comparison to existing nutritional prediction models in gastric or esophageal cancer (21), our study offers unique value by integrating GMM with machine learning to capture heterogeneous 12-month postoperative weight loss trajectories specific to gastric cancer survivorship, addressing gaps in prior work that relies on cross-sectional data or isolated machine learning approaches. Our findings reveal three distinct postoperative weight loss trajectories in patients with gastric cancer following radical gastrectomy, identified through GMM. The 3-class model exhibited superior fit, as evidenced by the lowest AIC and BIC values, statistically significant LMR-LRT and BLRT results, and high entropy, collectively confirming robust classification accuracy and model stability. Clinically, these trajectories—Severe (11.9%), Moderate (36.2%), and Minimal (51.9%) weight loss—demonstrated significantly divergent patterns over the 12-month follow-up period. This heterogeneity in nutritional recovery trajectories likely reflects individual variations in physiological responses to surgical stress and subsequent adaptive mechanisms. Critically, patients following moderate-to-severe weight loss trajectories constituted nearly half of the cohort (48.1%). This high proportion provides a plausible mechanistic explanation for the persistently elevated rates of malnutrition documented in previous studies of gastrectomy patients (6, 17, 35).

Furthermore, our analysis identified distinct predictors associated with membership in these specific weight loss trajectory groups. Severe weight loss (compared to minimal) was independently associated with older age, higher BMI, and total gastrectomy (vs. distal). Conversely, moderate weight loss (compared to severe) was linked to younger age, lower BMI, distal gastrectomy (vs. total), and not receiving adjuvant chemotherapy. Additionally, when compared to minimal weight loss, moderate weight loss showed stronger associations with female sex, advanced pathological stage, and postoperative major complications. These findings underscore the complex interplay between the extent of surgical resection, baseline patient characteristics, and tumor-related factors in determining postoperative nutritional outcomes. This evidence strongly supports the imperative for nutritional care strategies that are both individualized and informed by a patient’s predicted or observed weight loss trajectory.

Although postoperative weight loss constitutes a key phenotypic criterion within the GLIM framework, it is critical to recognize that weight loss and malnutrition are not synonymous concepts (36). For example, patients with pre-existing nutritional compromise (e.g., low BMI or CT-defined reduced muscle mass) may meet GLIM criteria for malnutrition even with minimal postoperative weight loss. Thus, weight loss primarily reflects a dynamic state of negative energy balance, while malnutrition represents a multifactorial syndrome characterized by compromised body composition, diminished physiological function, and impaired metabolic reserves (37, 38).

Developing a robust predictive model for postoperative malnutrition is clinically imperative. Early identification of high-risk patients enables timely, targeted nutritional interventions, which have been shown to reduce complications and hospital readmissions, enhance patient self-efficacy, alleviate cancer-related fatigue, improve adherence to self-care regimens, and ultimately improve long-term survival outcomes (16, 39, 40). The selection of the T2 for predictive modeling was driven by three key considerations: (1) T2 represented the peak prevalence of GLIM-defined malnutrition (51.6%) and coincided with maximal postoperative weight loss; (2) this timepoint aligns with institutional protocols for standard 6-month oncologic follow-up (including scheduled CT imaging and completion of adjuvant therapy), ensuring comprehensive data integration; (3) Earlier timepoints (e.g., T1) were confounded by transient nutritional instability during acute surgical recovery, potentially compromising diagnostic accuracy, whereas later assessments (T4) were susceptible to survivorship bias and higher attrition rates. Thus, T2 provides an optimal balance of clinical relevance, practical utility, and predictive validity.

Machine learning models have become indispensable in precision oncology, facilitating the integration of high-dimensional clinical data to uncover complex, non-linear relationships between predictors and outcomes (41). In this study, XGBoost demonstrated superior discriminative performance among all models, with optimal specificity, precision, and robust calibration, further enhanced by SHAP analysis for predictor interpretability. While SHAP provides clear insights into variable contributions within the machine learning framework, its implementation requires specialized software and integration into hospital electronic health record (EHR) systems, which may not be feasible in resource-limited settings. In contrast, the multivariable logistic regression-derived nomogram, despite a numerically lower AUC, showed statistically equivalent discrimination. The nomogram offers distinct clinical-translational advantages: (1) intuitive visualization of variable contributions for multidisciplinary teams; (2) immediate calculation of individualized risk probabilities via a user-friendly interface; and (3) point-of-care utility without requiring computational infrastructure, particularly through its dynamic, web-based version. These features make the nomogram uniquely suited for rapid risk stratification in diverse clinical environments, complementing the robust predictive power of machine learning models like XGBoost (42).

Precision nutritional management refers to tailored nutritional interventions based on individualized risk profiles from our XGBoost model and nomogram. A clinical pathway includes: (1) preoperative nutritional supplements (7–10 days) for high-risk patients (probability ≥0.56) to reduce complications (18); (2) early postoperative enteral feeding and high-protein diets; (3) multidisciplinary follow-up at 6 months with dietitian referrals and exercise programs. These steps, aligned with ESPEN guidelines (43), enhance clinical applicability.

Importantly, age ≥65 years was independently associated with both primary endpoints—GLIM-defined malnutrition and severe weight loss—aligning with known geriatric vulnerabilities. The aging process involves multisystem decline: attenuated anabolic hormone secretion, impaired muscle protein synthesis, and diminished adaptive capacity to metabolic stress. These alterations collectively compromise lean mass preservation and post-surgical recovery (44). Furthermore, age-associated comorbidities, anorexia, and dysregulated gastrointestinal motility synergistically exacerbate persistent nutrient deficits and malabsorptive syndromes post-gastrectomy (45, 46).

Our findings highlight distinct mechanisms linking preoperative BMI and reduced muscle mass to malnutrition risk at T2. Overweight patients (BMI ≥ 24 kg/m^2^) are prone to severe postoperative weight loss due to gastric reservoir loss and neurohormonal dysregulation, which drive caloric restriction and adipose tissue catabolism (17, 47). Conversely, underweight patients (BMI < 18.5 kg/m^2^) face higher GLIM-defined malnutrition risk due to low metabolic reserves and impaired capacity to meet surgical stress demands, exacerbated by postoperative maldigestion (18, 21). Similarly, preoperative reduced muscle mass independently predicts malnutrition by limiting amino acid availability for protein synthesis and correlating with systemic inflammation, which impairs nutrient utilization post-gastrectomy (48–50). These findings underscore the need for tailored nutritional strategies: intensive monitoring and supplementation for underweight patients pre- and postoperatively, and proactive management of rapid weight loss in overweight patients to prevent excessive depletion.

Total gastrectomy emerged as an independent risk factor for both malnutrition and severe postoperative weight loss. This finding aligns with the profound anatomical and physiological disruptions inherent to complete gastric resection. The absence of gastric reservoir capacity induces early satiety and markedly reduces caloric intake, while anatomical alterations (including duodenal exclusion and vagal denervation) drive malabsorption of critical nutrients—particularly vitamin B_12_, iron, and dietary fats (51–53). These mechanisms synergistically drive catabolic weight loss and nutritional depletion. Furthermore, the increased surgical invasiveness of total gastrectomy exacerbates systemic stress responses and inflammation, further depleting metabolic reserves and impeding adaptive recovery pathways (17, 54).

This study has several inherent limitations. First, this study’s single-center design may limit generalizability due to regional variations in surgical techniques, perioperative care, and patient characteristics influencing nutritional outcomes in gastric cancer. Standardized data collection ensures robust internal validity, but prospective multicenter validation is needed to confirm the XGBoost model’s and nomogram’s broader applicability. Second, the 12-month follow-up period precludes assessment of long-term nutritional sequelae beyond T4, such as osteoporosis or micronutrient deficiencies. Third, to ensure the integrity of the longitudinal trajectory analysis, we excluded patients with recurrence or mortality, who typically exhibit the most severe nutritional decline. This may introduce survivorship bias, potentially leading to an underestimation of malnutrition prevalence in the overall population. Finally, CT-dependent muscle mass assessment limits practical implementation in resource-constrained settings lacking routine imaging infrastructure.

## 5 Conclusion

This study identifies three distinct postoperative weight loss trajectories in gastric cancer patients after radical gastrectomy, with nearly half experiencing moderate-to-severe weight loss. We developed and validated both an XGBoost model and a nomogram that accurately predict 6-month postoperative malnutrition, with comparable discriminative power. These tools, informed by key factors such as age, preoperative BMI, muscle mass, and surgical type, provide a basis for personalized nutritional risk assessment and timely interventions to improve postoperative outcomes.

## Data Availability

The original contributions presented in the study are included in the article/Supplementary material, further inquiries can be directed to the corresponding author/s.

## Footnotes

^1^ https://ymfdoc.shinyapps.io/malnutrition/
