# Supplementary material for: Identification of postoperative weight loss trajectories and development of a machine learning-based tool for predicting malnutrition in gastric cancer patients
Source: Front Nutr. 2025 Sep 17;12:1678879. doi: 10.3389/fnut.2025.1678879 (PMC12483865; doi:10.3389/fnut.2025.1678879)
Supplement: Supplementary file 2 [file Table_1.DOC]

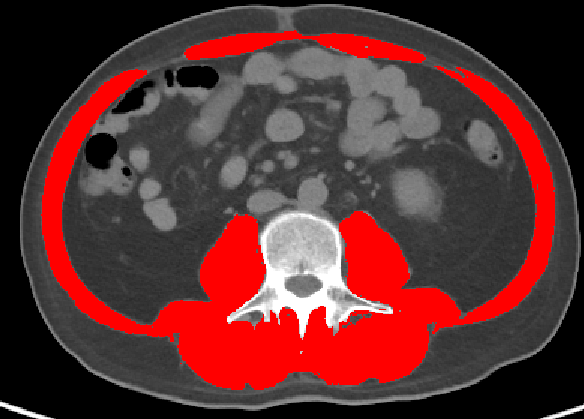


**SUPPLEMENTARY FIGURE 1**

Quantification of Skeletal Muscle Area (SMA) at L3 Level


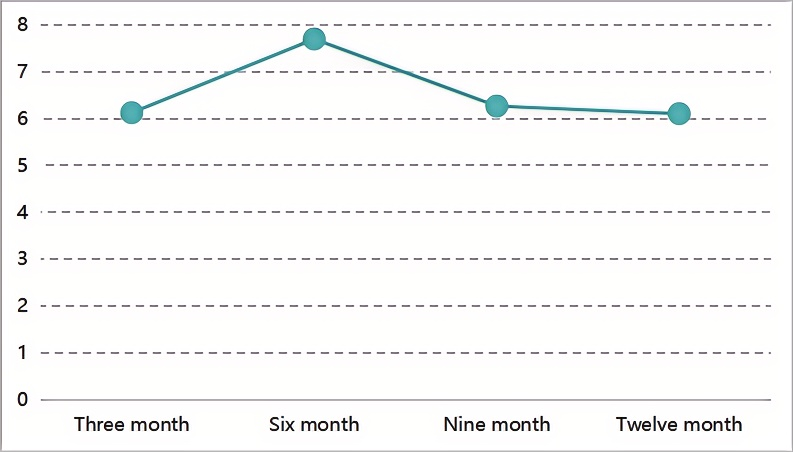


**SUPPLEMENTARY FIGURE 2**

Postoperative Weight Loss Percentage at Follow-up Intervals.


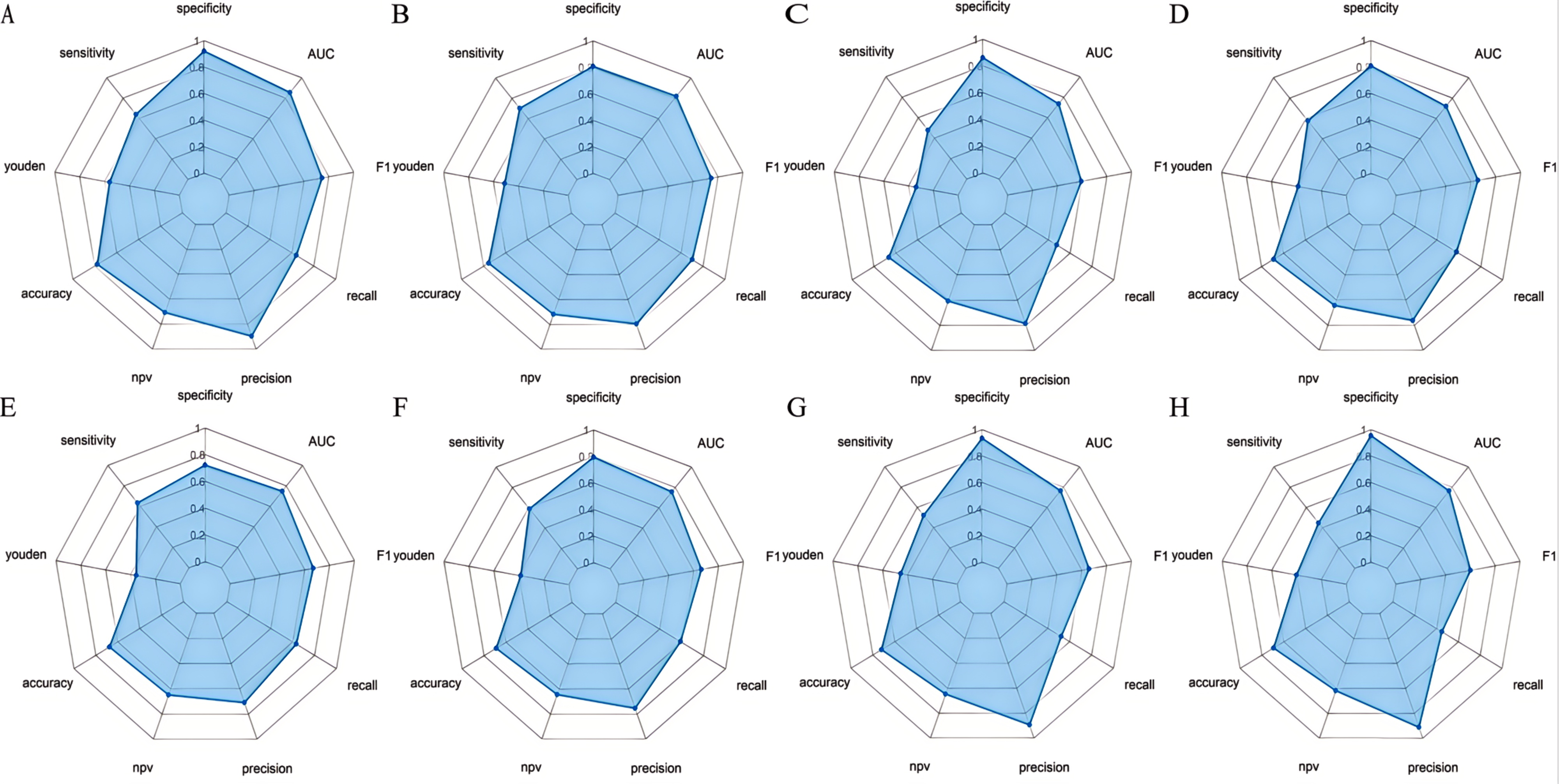


**SUPPLEMENTARY FIGURE** **3**

Radar-chart comparison of the eight machine-learning algorithms. (A)XGBoost; (B)SVM; (C)RF; (D)NB; (E)KNN; (F)MLP; (G)GBM; (H)PLS.
